# DNAmoreDB, a database of DNAzymes

**DOI:** 10.1093/nar/gkaa867

**Published:** 2020-10-14

**Authors:** Almudena Ponce-Salvatierra, Pietro Boccaletto, Janusz M Bujnicki

**Affiliations:** Laboratory of Bioinformatics and Protein Engineering, International Institute of Molecular and Cell Biology in Warsaw, ul. Ks. Trojdena 4, PL-02-109 Warsaw, Poland; Laboratory of Bioinformatics and Protein Engineering, International Institute of Molecular and Cell Biology in Warsaw, ul. Ks. Trojdena 4, PL-02-109 Warsaw, Poland; Laboratory of Bioinformatics and Protein Engineering, International Institute of Molecular and Cell Biology in Warsaw, ul. Ks. Trojdena 4, PL-02-109 Warsaw, Poland; Bioinformatics Laboratory, Institute of Molecular Biology and Biotechnology, Faculty of Biology, Adam Mickiewicz University, ul. Umultowska 89, PL-61-614 Poznan, Poland

## Abstract

Deoxyribozymes, DNA enzymes or simply DNAzymes are single-stranded oligo-deoxyribonucleotide molecules that, like proteins and ribozymes, possess the ability to perform catalysis. Although DNAzymes have not yet been found in living organisms, they have been isolated in the laboratory through in vitro selection. The selected DNAzyme sequences have the ability to catalyze a broad range of chemical reactions, utilizing DNA, RNA, peptides or small organic compounds as substrates. DNAmoreDB is a comprehensive database resource for DNAzymes that collects and organizes the following types of information: sequences, conditions of the selection procedure, catalyzed reactions, kinetic parameters, substrates, cofactors, structural information whenever available, and literature references. Currently, DNAmoreDB contains information about DNAzymes that catalyze 20 different reactions. We included a submission form for new data, a REST-based API system that allows users to retrieve the database contents in a machine-readable format, and keyword and BLASTN search features. The database is publicly available at https://www.genesilico.pl/DNAmoreDB/.

## Graphical Abstract

**Graphical Abstract:**
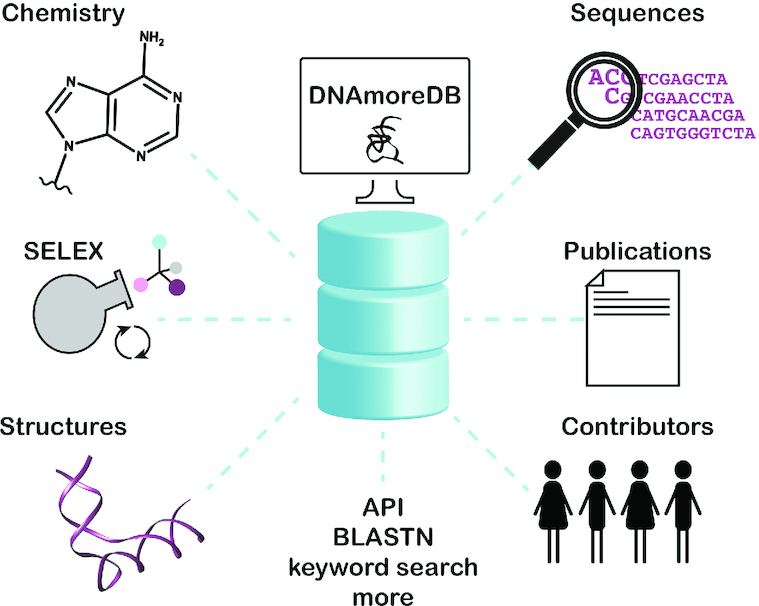
DNAmoreDB, a comprehensive database resource for DNAzymes.

## INTRODUCTION

DNAzymes, also known as deoxyribozymes, are synthetic single-stranded DNA molecules able to catalyze chemical reactions. Discovered in 1994 (1), this family of catalysts possesses desirable properties such as low cost and ease to synthesize, robustness, as well as adaptability and applicability to a wide range of processes alone or in combination with nanomaterials (2,3). DNAzymes known to date have been isolated by a process termed in vitro selection or SELEX (**S**ystematic **E**volution of **L**igands by **Ex**ponential enrichment) (4,5). This laboratory procedure mimics natural evolution at an accelerated pace and consists of rounds of selection that iteratively isolate DNAzymes from a random pool of sequences. The isolated sequences are amplified by the polymerase chain reaction after each round of isolation, while the population becomes enriched in sequences that exhibit the activity that is selected for. The selection experiment is concluded once the desired level of catalytic activity is reached, the active sequences are cloned and, typically, the most representative DNAzymes are characterized through functional assays.

Since the discovery of the first DNAzyme, a lead-dependent RNA-cleaving deoxyribozyme (1), hundreds of deoxyribozymes have been isolated, adding to an ever-growing repertoire of reactions that DNAzymes are able to catalyze. DNAzymes for RNA ligation (6), RNA cleavage (1,7), peptide side-chain modification (8,9), and DNA hydrolysis (9) are among the most pursued types of DNA catalysts. The underlying potential of DNAzymes has attracted scientists from very diverse fields, ultimately leading to their application as biosensors (10,11), therapeutic agents (12,13), and nanotechnology tools (14–16).

More than 1500 different DNAzyme sequences have been reported in the literature, accounting for at least 20 different catalytic activities. While deciphering the basis of DNA catalysis at the molecular level may seem to be still far in time, the wealth of biochemical data gathered throughout >25 years would serve the scientific community better if it is presented in an online resource, where it could be easy and intuitive to make comparisons, stimulate new ideas and foster data exchange and new collaborations. For instance, DNAzyme sequences are not normally available in the public sequence databases such as GenBank, with the exception of those that have had their 3D structure determined (17–21). Moreover, publications typically report 1 or 2 active sequences in the main text of the article. However, additional catalytic sequences are often reported in the supplementary information and, for this reason, are hard to retrieve for non-experts. We have developed the DNAmoreDB database as a single online resource to store and organize information about DNAzymes at different levels to facilitate their study, identify DNAzymes that already exist, and to enable the comparison of newly selected sequences to those in the database.

## DATABASE CONTENT

The current (as of 2020.09.21) version of DNAmoreDB contains 1782 sequences drawn from 116 published references (Supplementary data), and this dataset is expected to grow with the planned updates.

The *Home* page of DNAmoreDB introduces the database and provides quick links to several of its features. For instance, it is possible to query the database by keywords, see which pages can be browsed, and access the sequence search tool. The toolbar displays all the database's pages and a search bar to query the database contents.

**Figure 1.**
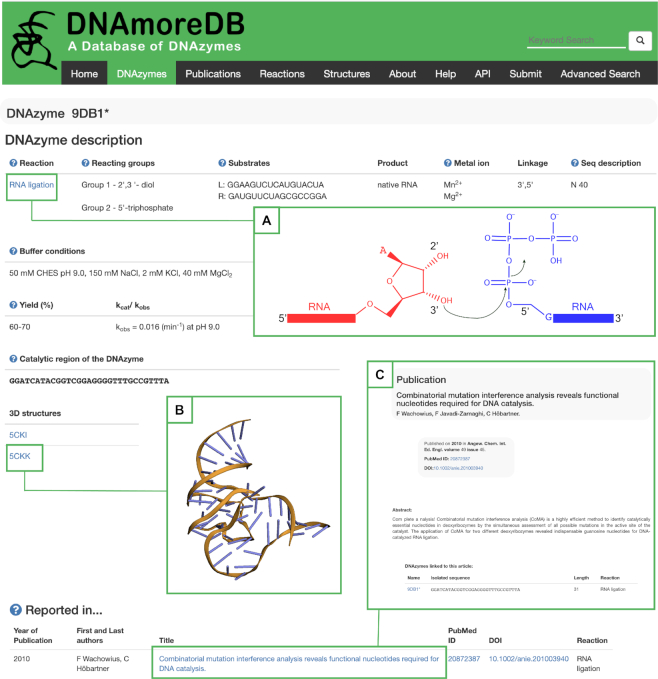
DNAmoreDB’s *Single entry* page. A summary of information collected for the DNAzyme 9DB1*, including links to the *Reactions* page (inset **A**), the *Structures* page (inset **B**), and the *Publications* page (inset **C**).

The DNAzymes present in the database are classified according to the reaction that they catalyze, namely: RNA cleavage (1,7), DNA cleavage (9,22), RNA ligation (6,23–25), DNA ligation (24,26), DNA site-specific depurination (27), Porphyrin metalation (28,29), DNA phosphorylation (30,31), DNA capping (32), amino acid side-chain modification (9,33,34), thymine dimer repair (35,36), Copper-mediated Azide-Alkyne Cycloaddition (CuAAC) (37), Dephosphorylation (38), Diels-Alder (39), Tyrosine azido‐adenylylation (40), Modification of Phosphorylated Amino Acid Side Chains (41,42), Tyrosine Phosphorylation (43,44), Glycosylation (45), Reductive amination (46), Amide hydrolysis (47,48), and Ester hydrolysis (48). This classification allows users to apply filters while browsing the *DNAzymes* page, which can be accessed from the toolbar and from the quick links on the *Home* page. DNAmoreDB recapitulates the data on DNAzymes available both in the main text of the publication as well as in the supplementary information. To avoid flooding a user with data, the ‘default’ option in the *DNAzymes* page shows only the DNAzymes reported in the main text of the article, unless otherwise specified by the user. The entries can be sorted according to different criteria, such as the length of the catalytic region or their name, and filters concerning the catalyzed reaction, the cofactor requirements, and whether or not the particular DNAzyme has been kinetically characterized can be applied to narrow down the results displayed. Moreover, the sequences displayed with bold characters indicate that the *single entry page* contains information about the reaction yield and/or rate constants. By clicking on a deoxyribozyme's name, the user is redirected to the *single entry* page (Figure 1), where the following information is displayed in a tabular format: DNAzyme's name, catalyzed reaction, substrate(s), reaction product(s), functional groups or residues taking part in the reaction, buffer conditions, yield and rate constant (if available), cofactors, the in vitro selected sequence and a *Notes* section. In addition, the primary reference is displayed, along with related publications, each showing first and last authors, title of the publication, PubMed ID, DOI, and the reaction for which the DNAzyme was selected. Within the *single entry* pages, the items displayed in blue are linked to other pages of the database or to external pages, while those containing an asterisk (*) point the user to the *Notes* section of the page. The internal pages of the database that can be accessed from single entry pages are the *Help*, *Reactions* (Figure 1A), *Structures* (Figure 1B), and *Publications* (Figure 1C); while the external pages that can be accessed are those of the RCSB database (49) if there is structural information available for a given entry, Pubmed, and scientific journals.

To have a broader view on selected DNAzymes, the user may browse the database from the *Publications* page, where the datasets reported in particular articles can be accessed by clicking on the publication title. In contrast to the DNAzymes’ *single entry* pages, in a publication's page, the available information comprises the publication's abstract, the authors list, the PubMed ID, the DOI, the name of the catalyzed reaction, the pool description, and the list of DNAzymes reported in it (Figure 1C). The *Publications* page and the single publication pages are also linked to internal and external resources, as are the *DNAzymes* and DNAzymes’ *single entry* pages.

A feature of DNAmoreDB useful for users interested in chemistry is the *Reactions* page. Accessible through the toolbar and by clicking on the reaction names (whenever they appear displayed in blue in other pages) aims at illustrating the reaction chemistries catalyzed by the DNAzymes contained in DNAmoreDB (Figure 1A). This page is not meant to be an exhaustive chemistry book, but rather to provide representative examples of how the deoxyribozyme's substrates may be activated for the reaction to take place.

Structural knowledge is critical to our understanding of DNA catalysis and crucial for advancing the research on DNAzymes and their possible technological applications. Many of the DNAzymes in DNAmoreDB have been thoroughly characterized from a biochemical and functional point of view, however, to date only the structures of two DNAzymes in functionally relevant conformations have been determined (18,19). The structures of these DNAzymes can be visualized under the *Structures* tab. For each DNAzyme, the PDB accession codes take the user to a *single structure page* (Figure 1B), where the DNAzyme 3D model is displayed along with relevant information such as the resolution at which the structure was determined, the method of structure determination, and the publication reporting the structure. Additionally, the PDB file can be directly downloaded from DNAmoreDB, although the user may as well follow the link to the original source of structural information (49).

The *Help* page walks the user through items linked to question mark icons. Whenever an item appears next to a blue question mark, the user can click on it and be redirected to the relevant section of the *Help* page in which additional information will be provided. Moreover, within this page, the user can consult a number of selected review papers to gain further knowledge on DNAzymes (Supplementary data).

The database is updated as new papers on the in vitro selection of DNAzymes become available. In particular, we continue to include literature references as they are brought to our attention. DNAmoreDB users are encouraged to use the contact form under the *Submit* tab to provide us with DNAzymes not yet included, with the scope of making DNAmoreDB as complete as possible.

## IMPLEMENTATION

DNAmoreDB has been implemented using Python v.3.6.9 (https://www.python.org/) programming language coupled with the Django web framework v.3.0.5 (http://www.djangoproject.com/) and the Apache2 HTTP server (https://httpd.apache.org/). The web server uses a PostgreSQL (https://www.postgresql.org/) relational database to store data and leverages on several open-source Javascript libraries as Datatables (http://datatables.net), to make the tables under *DNAzymes* and *Publications* pages sortable, interactive and searchable. Moreover, 3Dmol.js (50), a molecular data viewer, is used to show an interactive 3D rendering of DNAzymes structures, as deposited in the PDB within the *single structure pages*. The website is HTTPS-enabled, which means that the data exchange between the user and the DNAmoreDB server is secured by an encrypted connection. In addition, the website is mobile-friendly, adapting itself to the user's screen size and device, making DNAmoreDB easily accessible from tablets and smartphones.

A REST-based API was implemented to make the database contents available to external resources, such as other databases, in a machine-readable format. By simply manipulating the URL of DNAmoreDB it is possible to retrieve information about a single DNAzyme, a group of DNAzymes (e.g. RNA-ligating DNAzymes, or Mg^2+^-dependent DNAzymes), or all the DNAzymes stored in the database. The API system flattens the existing relations stored in the database, describing the DNAzyme data by using specific identifiers (Supplementary data Table S1). The data can be retrieved formatted as a JSON object (by default) or as CSV (comma-separated value) text. A detailed explanation on how to make use of the API along with several examples is available under the *API* tab of DNAmoreDB.

The advanced search page offers a possibility to query the database by keyword, sequence, DOI or PubMed ID. In addition to the content directly linked to textual matches (e.g. a DNAzyme name, a publication title, a reaction, etc.), the keyword search returns contents that are contextually related to the query. For example, if the user would like to retrieve information about the DNAzyme 9DB1, the keyword search ‘9DB1’ would not only retrieve the DNAzyme's single entry page, but also the article in which it was first reported, other relevant publications, and structural data. It is also possible to query the database by using the length of the random region used during the in vitro selection process. For example, using ‘N40’ as a keyword returns a list of DNAzymes that have a 40-nucleotide-long catalytic region.

The sequence search functionality has been implemented using NCBI Nucleotide-Nucleotide BLAST 2.4.0+ (BLASTN) (51,52), which allows querying the database with sequences in FASTA or RAW formats. The sequence search can be fine-tuned selecting the e-value threshold, below which the results should be presented (default is 1e-02) as well as the desired strand directionality (default is plus). The displayed results include the hits below the desired threshold listed according to the reactions they catalyze, their sequences, as well as the classical BLASTN output. The raw BLASTN results can be downloaded as a textual or JSON format.

## DISCUSSION

DNAmoreDB is dedicated to DNAzymes and it offers a single entry point for data that so far could be obtained only by meticulously analyzing many different sources, often difficult to browse, such as supplementary materials of published papers. Our database provides users with an easy-to-use interface with flexibility to browse from lists of DNAzymes and publications, filter the results according to different criteria, and choose the order in which the results are displayed. Additionally, advanced search options are available through keyword search, and by sequence.

DNAmoreDB is open for feedback from users to ensure that all DNAzymes’ published data are added, all errors are corrected, and up-to-date links to external databases are maintained. We encourage users to use the contact form under the *Submit* tab in the toolbar to report any error or malfunctioning of the database so that it can be fixed as soon as possible.

Future updates of the DNAmoreDB database will include a *Contributors* page, acknowledging all the groups contributing to the discovery and engineering of DNAzymes, and also an *Applications* page, that will make it possible to browse for DNAzymes and DNAzymes-based systems for practical applications. Although the current version of DNAmoreDB includes some reports in which dXTPs have been used, X being any nucleobase that has been modified to harbor a protein-like functionality, we have not included any DNAzymes with modified backbones (XNAs). XNAs and possibly other nucleic-acid based catalytic molecules will be included in the future releases of DNAmoreDB. In the next major update, we also plan to include predicted structures and sequence alignments for DNAzymes predicted to exhibit similar structures.

## DATA AVAILABILITY

The web interface to the database is available at https://www.genesilico.pl/DNAmoreDB/. This website is free, open to all users and no login or password is required.

## Supplementary Material

gkaa867_Supplemental_File

## References

[1] Breaker R.R., Joyce G.F. A DNA enzyme that cleaves RNA. Chem. Biol. 1994; 1:223–229. 9383394 10.1016/1074-5521(94)90014-0

[2] Silverman S.K. Catalytic DNA (deoxyribozymes) for synthetic applications—current abilities and future prospects. Chem. Commun. (Camb.). 2008; 2008:3467–3485. 10.1039/b807292m 18654692

[3] Morrison D., Rothenbroker M., Li Y. DNAzymes: selected for applications. Small Methods. 2018; 2:1700319.

[4] Tuerk C., Gold L. Systematic evolution of ligands by exponential enrichment: RNA ligands to bacteriophage T4 DNA polymerase. Science. 1990; 249:505–510. 2200121 10.1126/science.2200121

[5] Ellington A.D., Szostak J.W. In vitro selection of RNA molecules that bind specific ligands. Nature. 1990; 346:818–822. 1697402 10.1038/346818a0

[6] Purtha W.E., Coppins R.L., Smalley M.K., Silverman S.K. General deoxyribozyme-catalyzed synthesis of native 3′-5′ RNA linkages. J. Am. Chem. Soc. 2005; 127:13124–13125. 16173722 10.1021/ja0533702

[7] Santoro S.W., Joyce G.F. A general purpose RNA-cleaving DNA enzyme. Proc. Natl. Acad. Sci. U.S.A. 1997; 94:4262–4266. 9113977 10.1073/pnas.94.9.4262 PMC20710

[8] Wong O.Y., Pradeepkumar P.I., Silverman S.K. DNA-catalyzed covalent modification of amino acid side chains in tethered and free peptide substrates. Biochemistry. 2011; 50:4741–4749. 21510668 10.1021/bi200585n PMC3119496

[9] Velez T.E., Singh J., Xiao Y., Allen E.C., Wong O.Y., Chandra M., Kwon S.C., Silverman S.K. Systematic evaluation of the dependence of deoxyribozyme catalysis on random region length. ACS Comb. Sci. 2012; 14:680–687. 23088677 10.1021/co300111f PMC3518697

[10] Guo Y., Li J., Zhang X., Tang Y. A sensitive biosensor with a DNAzyme for lead(II) detection based on fluorescence turn-on. Analyst. 2015; 140:4642–4647. 25978496 10.1039/c5an00677e

[11] Ali M.M., Wolfe M., Tram K., Gu J., Filipe C.D.M., Li Y., Brennan J.D. A DNAzyme-based colorimetric paper sensor for Helicobacter pylori. Angew .Chem. Int. Ed. Engl. 2019; 58:9907–9911. 31095864 10.1002/anie.201901873

[12] Wang H., Chen Y., Wang H., Liu X., Zhou X., Wang F. DNAzyme-loaded metal-organic frameworks (MOFs) for self-sufficient gene therapy. Angew. Chem. Int. Ed. Engl. 2019; 58:7380–7384. 30916460 10.1002/anie.201902714

[13] Ali M.M., Slepenkin A., Peterson E., Zhao W. A simple DNAzyme-based fluorescent assay for Klebsiella pneumoniae. ChemBioChem. 2019; 20:906–910. 30521678 10.1002/cbic.201800701 PMC6692177

[14] Li F., Chen H., Pan J., Cha T.G., Medintz I.L., Choi J.H. A DNAzyme-mediated logic gate for programming molecular capture and release on DNA origami. Chem. Commun. (Camb). 2016; 52:8369–8372. 27211274 10.1039/c6cc02989b

[15] Liu J., Cui M., Zhou H., Yang W. DNAzyme based nanomachine for in situ detection of MicroRNA in living cells. ACS Sens. 2017; 2:1847–1853. 29181969 10.1021/acssensors.7b00710

[16] Zhu S., Wang X., Jing C., Yin Y., Zhou N. A colorimetric ATP assay based on the use of a magnesium(II)-dependent DNAzyme. Mikrochim. Acta. 2019; 186:176. 30771011 10.1007/s00604-019-3244-9

[17] NCBI Resource Coordinators. Database resources of the National Center for Biotechnology Information. Nucleic Acids Res. 2013; 41:D8–D20. 23193264 10.1093/nar/gks1189 PMC3531099

[18] Ponce-Salvatierra A., Wawrzyniak-Turek K., Steuerwald U., Höbartner C., Pena V. Crystal structure of a DNA catalyst. Nature. 2016; 529:231–234. 26735012 10.1038/nature16471

[19] Liu H., Yu X., Chen Y., Zhang J., Wu B., Zheng L., Haruehanroengra P., Wang R., Li S., Lin J. et al. Crystal structure of an RNA-cleaving DNAzyme. Nat. Commun. 2017; 8:2006. 29222499 10.1038/s41467-017-02203-x PMC5722873

[20] Nowakowski J., Shim P., Prasad G., Stout C.D., Joyce G.F. Crystal structure of an 82-nucleotide RNA–DNA complex formed by the 10–23 DNA enzyme. Nat. Struct. Mol. Biol. 1999; 6:151–156. 10.1038/5839 10048927

[21] Nowakowski J., Shim P.J., Stout C.D., Joyce G.F. Alternative conformations of a nucleic acid four-way junction. J. Mol. Biol. 2000; 300:93–102. 10864501 10.1006/jmbi.2000.3826

[22] Gu H., Furukawa K., Weinberg Z., Berenson D.F., Breaker R.R. Small, highly active DNAs that hydrolyze DNA. J. Am. Chem. Soc. 2013; 135:9121–9129. 23679108 10.1021/ja403585e PMC3763483

[23] Coppins R.L., Silverman S.K. A DNA enzyme that mimics the first step of RNA splicing. Nat. Struct. Mol. Biol. 2004; 11:270–274. 14758353 10.1038/nsmb727

[24] Lee C.S., Mui T.P., Silverman S.K. Improved deoxyribozymes for synthesis of covalently branched DNA and RNA. Nucleic Acids Res. 2011; 39:269–279. 20739352 10.1093/nar/gkq753 PMC3017605

[25] Purtha W.E., Coppins R.L., Smalley M.K., Silverman S.K. General deoxyribozyme-catalyzed synthesis of native 3′-5′ RNA linkages. J. Am. Chem. Soc. 2005; 127:13124–13125. 16173722 10.1021/ja0533702

[26] Cuenoud B., Szostak J.W. A DNA metalloenzyme with DNA ligase activity. Nature. 1995; 375:611–614. 7791880 10.1038/375611a0

[27] Höbartner C., Pradeepkumar P.I., Silverman S.K. Site-selective depurination by a periodate-dependent deoxyribozyme. Chem. Commun. (Camb). 2007; 2255–2257. 17534508 10.1039/b704507g

[28] Li Y., Sen D. A catalytic DNA for porphyrin metallation. Nat. Struct. Biol. 1996; 3:743–747. 8784345 10.1038/nsb0996-743

[29] Yang H., Peng D., Zhou Y., Liu J. Pb^2+^ as a substrate and a cofactor of a porphyrin metalation DNAzyme. Chembiochem. 2020; 21:2259–2263. 32202058 10.1002/cbic.202000073

[30] Wang W., Billen L.P., Li Y. Sequence diversity, metal specificity, and catalytic proficiency of metal-dependent phosphorylating DNA enzymes. Chem. Biol. 2002; 9:507–517. 11983339 10.1016/s1074-5521(02)00127-8

[31] Camden A.J., Walsh S.M., Suk S.H., Silverman S.K. DNA Oligonucleotide 3′-Phosphorylation by a DNA Enzyme. Biochemistry. 2016; 55:2671–2676. 27063020 10.1021/acs.biochem.6b00151 PMC4862906

[32] Li Y., Liu Y., Breaker R.R. Capping DNA with DNA. Biochemistry. 2000; 39:3106–3114. 10715132 10.1021/bi992710r

[33] Pradeepkumar P.I., Höbartner C., Baum D.A., Silverman S.K. DNA-catalyzed formation of nucleopeptide linkages. Angew. Chem. Int. Ed. Engl. 2008; 47:1753–1757. 18214866 10.1002/anie.200703676

[34] Wong O.Y., Pradeepkumar P.I., Silverman S.K. DNA-catalyzed covalent modification of amino acid side chains in tethered and free peptide substrates. Biochemistry. 2011; 50:4741–4749. 21510668 10.1021/bi200585n PMC3119496

[35] Chinnapen D.J., Sen D. A deoxyribozyme that harnesses light to repair thymine dimers in DNA. Proc. Natl. Acad. Sci. U.S.A. 2004; 101:65–69. 14691255 10.1073/pnas.0305943101 PMC314139

[36] Thorne R.E., Chinnapen D.J., Sekhon G.S., Sen D. A deoxyribozyme, Sero1C, uses light and serotonin to repair diverse pyrimidine dimers in DNA. J. Mol. Biol. 2009; 388:21–29. 19281822 10.1016/j.jmb.2009.02.064

[37] Liu K., Lat P.K., Yu H.Z., Sen D. CLICK-17, a DNA enzyme that harnesses ultra-low concentrations of either Cu+ or Cu2+ to catalyze the azide-alkyne 'click' reaction in water. Nucleic. Acids. Res. 2020; 48:7356–7370. 32520335 10.1093/nar/gkaa502 PMC7367168

[38] Chandrasekar J., Silverman S.K. Catalytic DNA with phosphatase activity. Proc. Natl. Acad. Sci. U.S.A. 2013; 110:5315–5320. 23509279 10.1073/pnas.1221946110 PMC3619321

[39] Chandra M., Silverman S.K. DNA and RNA can be equally efficient catalysts for carbon-carbon bond formation. J. Am. Chem. Soc. 2008; 130:2936–2937. 18271591 10.1021/ja7111965

[40] Wang P., Silverman S.K. DNA-catalyzed introduction of Azide at tyrosine for peptide modification. Angew. Chem. Int. Ed. Engl. 2016; 55:10052–10056. 27391404 10.1002/anie.201604364 PMC4993102

[41] Sachdeva A., Chandra M., Chandrasekar J., Silverman S.K. Covalent tagging of phosphorylated peptides by phosphate-specific deoxyribozymes. ChemBioChem. 2012; 13:654–657. 22315198 10.1002/cbic.201200048 PMC3306493

[42] Chandrasekar J., Wylder A.C., Silverman S.K. Phosphoserine lyase deoxyribozymes: DNA-catalyzed formation of dehydroalanine residues in peptides. J. Am. Chem. Soc. 2015; 137:9575–9578. 26200899 10.1021/jacs.5b06308 PMC4527949

[43] Walsh S.M., Sachdeva A., Silverman S.K. DNA catalysts with tyrosine kinase activity. J. Am. Chem. Soc. 2013; 135:14928–14931. 24066831 10.1021/ja407586u PMC3832193

[44] Walsh S.M., Konecki S.N., Silverman S.K. Identification of sequence-selective tyrosine kinase deoxyribozymes. J. Mol. Evol. 2015; 81:218–224. 26407964 10.1007/s00239-015-9699-3 PMC4662894

[45] Hesser A.R., Brandsen B.M., Walsh S.M., Wang P., Silverman S.K. DNA-catalyzed glycosylation using aryl glycoside donors. Chem Commun (Camb). 2016; 52:9259–9262. 27355482 10.1039/c6cc04329a PMC4945402

[46] Wong O.Y., Mulcrone A.E., Silverman S.K. DNA-catalyzed reductive amination. Angew. Chem. Int. Ed. Engl. 2011; 50:11679–11684. 21994131 10.1002/anie.201104976 PMC3269127

[47] Zhou C., Avins J.L., Klauser P.C., Brandsen B.M., Lee Y., Silverman S.K. DNA-catalyzed amide hydrolysis. J. Am. Chem. Soc. 2016; 138:2106–2109. 26854515 10.1021/jacs.5b12647 PMC4767666

[48] Brandsen B.M., Hesser A.R., Castner M.A., Chandra M., Silverman S.K. DNA-catalyzed hydrolysis of esters and aromatic amides. J. Am. Chem. Soc. 2013; 135:16014–16017. 24127695 10.1021/ja4077233 PMC3946404

[49] Berman H.M., Westbrook J., Feng Z., Gilliland G., Bhat T.N., Weissig H., Shindyalov I.N., Bourne P.E. The Protein Data Bank. Nucleic Acids Res. 2000; 28:235–242. 10592235 10.1093/nar/28.1.235 PMC102472

[50] Rego N., Koes D. 3Dmol.js: molecular visualization with WebGL. Bioinformatics. 2015; 31:1322–1324. 25505090 10.1093/bioinformatics/btu829 PMC4393526

[51] Altschul S.F., Gish W., Miller W., Myers E.W., Lipman D.J. Basic local alignment search tool. J. Mol. Biol. 1990; 215:403–410. 2231712 10.1016/S0022-2836(05)80360-2

[52] Camacho C., Coulouris G., Avagyan V., Ma N., Papadopoulos J., Bealer K., Madden T.L. BLAST+: architecture and applications. BMC Bioinformatics. 2009; 10:421. 20003500 10.1186/1471-2105-10-421 PMC2803857

