# Supplementary material for: DNAmoreDB, a database of DNAzymes
Source: Nucleic Acids Res. 2020 Oct 14;49(D1):D76–81. doi: 10.1093/nar/gkaa867 (PMC7778931; doi:10.1093/nar/gkaa867)
Supplement: gkaa867_Supplemental_File [file gkaa867_supplemental_file.pdf]

Supplementary data

DNAmoreDB, a database of DNAzymes.

Almudena Ponce-Salvatierra[1,*], Pietro Boccaletto[1], Janusz M. Bujnicki[1,2,*].

1 Laboratory of Bioinformatics and Protein Engineering, International Institute of Molecular and Cell Biology in Warsaw, ul. Ks. Trojdena 4, PL-02-109 Warsaw, Poland;
2 Bioinformatics Laboratory, Institute of Molecular Biology and Biotechnology, Faculty of Biology, Adam Mickiewicz University, ul. Umultowska 89, PL-61-614 Poznan, Poland;

* To whom correspondence should be addressed. Tel: (+48-22) 597-07-50; Fax: (+48-22) 597-07-15; Email: aponces@genesilico.pl.
Correspondence may also be addressed to Janusz M. Bujnicki: iamb@genesilico.pl

Table of contents:

References from which the content of the database has been retrieved…………………….....1
References included in the Help page of DNAmoreDB (Reviews)...8
Table S1. JSON/CSV identifiers…………………………………………………………………...10

References from which the content of the database has been retrieved.

1. Saran, R., & Liu, J. (2016). A Silver DNAzyme. *Analytical chemistry*, *88*(7), 4014–4020.
2. Faulhammer, D., & Famulok, M. (1997). Characterization and divalent metal-ion dependence of in vitro selected deoxyribozymes which cleave DNA/RNA chimeric oligonucleotides. *Journal of molecular biology*, *269*(2), 188–202.
3. Breaker, R. R., & Joyce, G. F. (1994). A DNA enzyme that cleaves RNA. *Chemistry & biology*, *1*(4), 223–229.
4. Geyer, C. R., & Sen, D. (1997). Evidence for the metal-cofactor independence of an RNA phosphodiester-cleaving DNA enzyme. *Chemistry & biology*, *4*(8), 579–593.
5. Liu, Z., Mei, S. H., Brennan, J. D., & Li, Y. (2003). Assemblage of signaling DNA enzymes with intriguing metal-ion specificities and pH dependences. *Journal of the American Chemical Society*, *125*(25), 7539–7545.
6. Parker, D. J., Xiao, Y., Aguilar, J. M., & Silverman, S. K. (2013). DNA catalysis of a normally disfavored RNA hydrolysis reaction. *Journal of the American Chemical Society*, *135*(23), 8472–8475.
7. Faulhammer D, & Famulok M. (1996). The Ca2+ Ion as a Cofactor for a Novel RNA-Cleaving Deoxyribozyme. Angewandte Chemie International Edition, 35(23-24), 2837-2841.
8. Li, J., Zheng, W., Kwon, A. H., & Lu, Y. (2000). In vitro selection and characterization of a highly efficient Zn(II)-dependent RNA-cleaving deoxyribozyme. *Nucleic acids research*, *28*(2), 481–488.
9. Breaker, R. R., & Joyce, G. F. (1995). A DNA enzyme with Mg(2+)-dependent RNA phosphoesterase activity. *Chemistry & biology*, *2*(10), 655–660.
10. Roth, A., & Breaker, R. R. (1998). An amino acid as a cofactor for a catalytic polynucleotide. *Proceedings of the National Academy of Sciences of the United States of America*, *95*(11), 6027–6031.
11. Kasprowicz, A., Stokowa-Sołtys, K., Jeżowska-Bojczuk, M., Wrzesiński, J., & Ciesiołka, J. (2016). Characterization of Highly Efficient RNA-Cleaving DNAzymes that Function at Acidic pH with No Divalent Metal-Ion Cofactors. *ChemistryOpen*, *6*(1), 46–56.
12. Liaqat, A., Stiller, C., Michel, M., Sednev, M. V., & Höbartner, C. (2020). N 6-isopentenyladenosine in RNA determines the cleavage site of endonuclease deoxyribozymes. *Angewandte Chemie (International ed. in English)*, 10.1002/anie.202006218. Advance online publication.
13. Sednev, M. V., Mykhailiuk, V., Choudhury, P., Halang, J., Sloan, K. E., Bohnsack, M. T., & Höbartner, C. (2018). N6 -Methyladenosine-Sensitive RNA-Cleaving Deoxyribozymes. *Angewandte Chemie (International ed. in English)*, *57*(46), 15117–15121.
14. Lee, Y., Klauser, P. C., Brandsen, B. M., Zhou, C., Li, X., & Silverman, S. K. (2017). DNA-Catalyzed DNA Cleavage by a Radical Pathway with Well-Defined Products. *Journal of the American Chemical Society*, *139*(1), 255–261.
15. Carmi, N., Shultz, L. A., & Breaker, R. R. (1996). In vitro selection of self-cleaving DNAs. *Chemistry & biology*, *3*(12), 1039–1046.
16. Velez, T. E., Singh, J., Xiao, Y., Allen, E. C., Wong, O. Y., Chandra, M., Kwon, S. C., & Silverman, S. K. (2012). Systematic evaluation of the dependence of deoxyribozyme catalysis on random region length. *ACS combinatorial science*, *14*(12), 680–687.

17. Chandra, M., Sachdeva, A., & Silverman, S. K. (2009). DNA-catalyzed sequence-specific hydrolysis of DNA. *Nature chemical biology*, *5*(10), 718–720.
18. Xiao, Y., Wehrmann, R. J., Ibrahim, N. A., & Silverman, S. K. (2012). Establishing broad generality of DNA catalysts for site-specific hydrolysis of single-stranded DNA. *Nucleic acids research*, *40*(4), 1778–1786.
19. Xiao, Y., Chandra, M., & Silverman, S. K. (2010). Functional compromises among pH tolerance, site specificity, and sequence tolerance for a DNA-hydrolyzing deoxyribozyme. *Biochemistry*, *49*(44), 9630–9637.
20. Dokukin, V., & Silverman, S. K. (2012). Lanthanide ions as required cofactors for DNA catalysts. *Chemical science*, *3*(5), 1707–1714.
21. Wong, O. Y., Pradeepkumar, P. I., & Silverman, S. K. (2011). DNA-catalyzed covalent modification of amino acid side chains in tethered and free peptide substrates. *Biochemistry*, *50*(21), 4741–4749.
22. Brandsen, B. M., Velez, T. E., Sachdeva, A., Ibrahim, N. A., & Silverman, S. K. (2014). DNA-catalyzed lysine side chain modification. *Angewandte Chemie (International ed. in English)*, *53*(34), 9045–9050.
23. Pradeepkumar, P. I., Höbartner, C., Baum, D. A., & Silverman, S. K. (2008). DNA-catalyzed formation of nucleopeptide linkages. *Angewandte Chemie (International ed. in English)*, *47*(9), 1753–1757.
24. Sachdeva, A., & Silverman, S. K. (2012). DNA-catalyzed reactivity of a phosphoramidate functional group and formation of an unusual pyrophosphoramidate linkage. *Organic & biomolecular chemistry*, *10*(1), 122–125.
25. Chu, C., Wong, O. Y., & Silverman, S. K. (2014). A generalizable DNA-catalyzed approach to peptide-nucleic acid conjugation. *Chembiochem : a European journal of chemical biology*, *15*(13), 1905–1910.
26. Sachdeva, A., & Silverman, S. K. (2010). DNA-catalyzed serine side chain reactivity and selectivity. *Chemical communications (Cambridge, England)*, *46*(13), 2215–2217.
27. Thorne, R. E., Chinnapen, D. J., Sekhon, G. S., & Sen, D. (2009). A deoxyribozyme, Sero1C, uses light and serotonin to repair diverse pyrimidine dimers in DNA. *Journal of molecular biology*, *388*(1), 21–29.
28. Chinnapen, D. J., & Sen, D. (2004). A deoxyribozyme that harnesses light to repair thymine dimers in DNA. *Proceedings of the National Academy of Sciences of the United States of America*, *101*(1), 65–69.
29. Yang, J., & Bowser, M. T. (2013). Capillary electrophoresis-SELEX selection of catalytic DNA aptamers for a small-molecule porphyrin target. *Analytical chemistry*, *85*(3), 1525–1530.
30. Li, Y., & Sen, D. (1996). A catalytic DNA for porphyrin metallation. *Nature structural biology*, *3*(9), 743–747.
31. Peng, D., Li, Y., Huang, Z., Liang, R. P., Qiu, J. D., & Liu, J. (2019). Efficient DNA-Catalyzed Porphyrin Metalation for Fluorescent Ratiometric Pb2+ Detection. *Analytical chemistry*, *91*(17), 11403–11408.
32. Li, Y., & Sen, D. (1997). Toward an efficient DNAzyme. *Biochemistry*, *36*(18), 5589–5599.
33. Camden, A. J., Walsh, S. M., Suk, S. H., & Silverman, S. K. (2016). DNA Oligonucleotide 3'-Phosphorylation by a DNA Enzyme. *Biochemistry*, *55*(18), 2671–2676.

34. Wang, W., Billen, L. P., & Li, Y. (2002). Sequence diversity, metal specificity, and catalytic proficiency of metal-dependent phosphorylating DNA enzymes. *Chemistry & biology*, *9*(4), 507–517.
35. Li, Y., Liu, Y., & Breaker, R. R. (2000). Capping DNA with DNA. *Biochemistry*, *39*(11), 3106–3114.
36. Mui, T. P., & Silverman, S. K. (2008). Convergent and general one-step DNA-catalyzed synthesis of multiply branched DNA. *Organic letters*, *10*(20), 4417–4420.
37. Wang, Y., & Silverman, S. K. (2003). Deoxyribozymes that synthesize branched and lariat RNA. *Journal of the American Chemical Society*, *125*(23), 6880–6881. https://doi.org/10.1021/ja035150z
38. Semlow, D. R., & Silverman, S. K. (2005). Parallel selections in vitro reveal a preference for 2'-5' RNA ligation upon deoxyribozyme-mediated opening of a 2',3'-cyclic phosphate. *Journal of molecular evolution*, *61*(2), 207–215.
39. Pratico, E. D., Wang, Y., & Silverman, S. K. (2005). A deoxyribozyme that synthesizes 2',5'-branched RNA with any branch-site nucleotide. *Nucleic acids research*, *33*(11), 3503–3512.
40. Coppins, R. L., & Silverman, S. K. (2004). A DNA enzyme that mimics the first step of RNA splicing. *Nature structural & molecular biology*, *11*(3), 270–274.
41. Behera, A. K., Schlund, K. J., Mason, A. J., Alila, K. O., Han, M., Grout, R. L., & Baum, D. A. (2013). Enhanced deoxyribozyme-catalyzed RNA ligation in the presence of organic cosolvents. *Biopolymers*, *99*(6), 382–391.
42. Prior, T. K., Semlow, D. R., Flynn-Charlebois, A., Rashid, I., & Silverman, S. K. (2004). Structure-function correlations derived from faster variants of a RNA ligase deoxyribozyme. *Nucleic acids research*, *32*(3), 1075–1082.
43. Kost, D. M., Gerdt, J. P., Pradeepkumar, P. I., & Silverman, S. K. (2008). Controlling the direction of site-selectivity and regioselectivity in RNA ligation by Zn2+-dependent deoxyribozymes that use 2',3'-cyclic phosphate RNA substrates. *Organic & biomolecular chemistry*, *6*(23), 4391–4398.
44. Lee, C. S., Mui, T. P., & Silverman, S. K. (2011). Improved deoxyribozymes for synthesis of covalently branched DNA and RNA. Nucleic acids research, 39(1), 269–279.
45. Wachowius, F., Javadi-Zarnaghi, F., & Höbartner, C. (2010). Combinatorial mutation interference analysis reveals functional nucleotides required for DNA catalysis. *Angewandte Chemie (International ed. in English)*, *49*(45), 8504–8508.
46. Wang, Y., & Silverman, S. K. (2005). Directing the outcome of deoxyribozyme selections to favor native 3'-5' RNA ligation. *Biochemistry*, *44*(8), 3017–3023.
47. Zelin, E., Wang, Y., & Silverman, S. K. (2006). Adenosine is inherently favored as the branch-site RNA nucleotide in a structural context that resembles natural RNA splicing. *Biochemistry*, *45*(9), 2767–2771.
48. Purtha, W. E., Coppins, R. L., Smalley, M. K., & Silverman, S. K. (2005). General deoxyribozyme-catalyzed synthesis of native 3'-5' RNA linkages. *Journal of the American Chemical Society*, *127*(38), 13124–13125.
49. Paul, N., Springsteen, G., & Joyce, G. F. (2006). Conversion of a ribozyme to a deoxyribozyme through in vitro evolution. *Chemistry & biology*, *13*(3), 329–338.
50. Coppins, R. L., & Silverman, S. K. (2004). Rational modification of a selection strategy leads to deoxyribozymes that create native 3'-5' RNA linkages. *Journal of the American Chemical Society*, *126*(50), 16426–16432.

51. Flynn-Charlebois, A., Wang, Y., Prior, T. K., Rashid, I., Hoadley, K. A., Coppins, R. L., Wolf, A. C., & Silverman, S. K. (2003). Deoxyribozymes with 2'-5' RNA ligase activity. *Journal of the American Chemical Society*, *125*(9), 2444–2454.
52. Flynn-Charlebois, A., Prior, T. K., Hoadley, K. A., & Silverman, S. K. (2003). In vitro evolution of an RNA-cleaving DNA enzyme into an RNA ligase switches the selectivity from 3'-5' to 2'-5'. *Journal of the American Chemical Society*, *125*(18), 5346–5350.
53. Hoadley, K. A., Purtha, W. E., Wolf, A. C., Flynn-Charlebois, A., & Silverman, S. K. (2005). Zn2+-dependent deoxyribozymes that form natural and unnatural RNA linkages. *Biochemistry*, *44*(25), 9217–9231.
54. Cuenoud, B., & Szostak, J. W. (1995). A DNA metalloenzyme with DNA ligase activity. *Nature*, *375*(6532), 611–614.
55. Liu, K., Lat, P. K., Yu, H. Z., & Sen, D. (2020). CLICK-17, a DNA enzyme that harnesses ultra-low concentrations of either Cu+ or Cu2+ to catalyze the azide-alkyne 'click' reaction in water. *Nucleic acids research*, *48*(13), 7356–7370.
56. Chandrasekar, J., & Silverman, S. K. (2013). Catalytic DNA with phosphatase activity. *Proceedings of the National Academy of Sciences of the United States of America*, *110*(14), 5315–5320.
57. Brandsen, B. M., Hesser, A. R., Castner, M. A., Chandra, M., & Silverman, S. K. (2013). DNA-catalyzed hydrolysis of esters and aromatic amides. *Journal of the American Chemical Society*, *135*(43), 16014–16017.
58. Zhou, C., Avins, J. L., Klauser, P. C., Brandsen, B. M., Lee, Y., & Silverman, S. K. (2016). DNA-Catalyzed Amide Hydrolysis. *Journal of the American Chemical Society*, *138*(7), 2106–2109.
59. Wong, O. Y., Mulcrone, A. E., & Silverman, S. K. (2011). DNA-catalyzed reductive amination. *Angewandte Chemie (International ed. in English)*, *50*(49), 11679–11684.
60. Hesser, A. R., Brandsen, B. M., Walsh, S. M., Wang, P., & Silverman, S. K. (2016). DNA-catalyzed glycosylation using aryl glycoside donors. *Chemical communications (Cambridge, England)*, *52*(59), 9259–9262.
61. Walsh, S. M., Sachdeva, A., & Silverman, S. K. (2013). DNA catalysts with tyrosine kinase activity. *Journal of the American Chemical Society*, *135*(40), 14928–14931.
62. Walsh, S. M., Konecki, S. N., & Silverman, S. K. (2015). Identification of Sequence-Selective Tyrosine Kinase Deoxyribozymes. *Journal of molecular evolution*, *81*(5-6), 218–224.
63. Dokukin, V., & Silverman, S. K. (2014). A modular tyrosine kinase deoxyribozyme with discrete aptamer and catalyst domains. *Chemical communications (Cambridge, England)*, *50*(66), 9317–9320.
64. Chandrasekar, J., Wylder, A. C., & Silverman, S. K. (2015). Phosphoserine Lyase Deoxyribozymes: DNA-Catalyzed Formation of Dehydroalanine Residues in Peptides. *Journal of the American Chemical Society*, *137*(30), 9575–9578. https://doi.org/10.1021/jacs.5b06308
65. Sachdeva, A., Chandra, M., Chandrasekar, J., & Silverman, S. K. (2012). Covalent tagging of phosphorylated peptides by phosphate-specific deoxyribozymes. *Chembiochem : a European journal of chemical biology*, *13*(5), 654–657.
66. Wang, P., & Silverman, S. K. (2016). DNA-Catalyzed Introduction of Azide at Tyrosine for Peptide Modification. *Angewandte Chemie (International ed. in English)*, *55*(34), 10052–10056.

67. Chandra, M., & Silverman, S. K. (2008). DNA and RNA can be equally efficient catalysts for carbon-carbon bond formation. *Journal of the American Chemical Society*, *130*(10), 2936–2937.
68. Höbartner, C., Pradeepkumar, P. I., & Silverman, S. K. (2007). Site-selective depurination by a periodate-dependent deoxyribozyme. *Chemical communications (Cambridge, England)*, (22), 2255–2257.
69. Sheppard, T. L., Ordoukhanian, P., & Joyce, G. F. (2000). A DNA enzyme with N-glycosylase activity. *Proceedings of the National Academy of Sciences of the United States of America*, *97*(14), 7802–7807.
70. Huang, P. J., Vazin, M., & Liu, J. (2014). In vitro selection of a new lanthanide-dependent DNAzyme for ratiometric sensing lanthanides. *Analytical chemistry*, *86*(19), 9993–9999.
71. Torabi, S. F., Wu, P., McGhee, C. E., Chen, L., Hwang, K., Zheng, N., Cheng, J., & Lu, Y. (2015). In vitro selection of a sodium-specific DNAzyme and its application in intracellular sensing. *Proceedings of the National Academy of Sciences of the United States of America*, *112*(19), 5903–5908.
72. Gu, H., Furukawa, K., Weinberg, Z., Berenson, D. F., & Breaker, R. R. (2013). Small, highly active DNAs that hydrolyze DNA. *Journal of the American Chemical Society*, *135*(24), 9121–9129.
73. Hollenstein, M., Hipolito, C., Lam, C., Dietrich, D., & Perrin, D. M. (2008). A highly selective DNAzyme sensor for mercuric ions. *Angewandte Chemie* (International ed. in English), 47(23), 4346–4350.
74. Liu, J., Brown, A. K., Meng, X., Cropek, D. M., Istok, J. D., Watson, D. B., & Lu, Y. (2007). A catalytic beacon sensor for uranium with parts-per-trillion sensitivity and millionfold selectivity. *Proceedings of the National Academy of Sciences of the United States of America*, *104*(7), 2056–2061.
75. Wang, Y., Yang, J., Yuan, X. *et al.* A Novel Small RNA-Cleaving Deoxyribozyme with a Short Binding Arm. *Sci Rep* 9, 8224 (2019)
76. Hollenstein, M., Hipolito, C. J., Lam, C. H., & Perrin, D. M. (2013). Toward the combinatorial selection of chemically modified DNAzyme RNase A mimics active against all-RNA substrates. *ACS combinatorial science*, *15*(4), 174–182.
77. Hollenstein, M., Hipolito, C. J., Lam, C. H., & Perrin, D. M. (2009). A self-cleaving DNA enzyme modified with amines, guanidines and imidazoles operates independently of divalent metal cations (M2+). *Nucleic acids research*, 37(5), 1638–1649.
78. Nelson, K. E., Bruesehoff, P. J., & Lu, Y. (2005). In vitro selection of high temperature Zn(2+)-dependent DNAzymes. *Journal of molecular evolution*, 61(2), 216–225.
79. Scheitl, C., Lange, S., & Höbartner, C. (2020). New Deoxyribozymes for the Native Ligation of RNA. Molecules (Basel, Switzerland), 25(16), E3650.
80. Li, Y., & Breaker, R. R. (1999). Phosphorylating DNA with DNA. *Proceedings of the National Academy of Sciences of the United States of America*, 96(6), 2746–2751.
81. Gu, L., Saran, R., Yan, W., Huang, P. J., Wang, S., Lyu, M., & Liu, J. (2018). Reselection Yielding a Smaller and More Active Silver-Specific DNAzyme. *ACS omega*, 3(11), 15174–15181.
82. Ma, L., & Liu, J. (2019). An in Vitro-Selected DNAzyme Mutant Highly Specific for Na+ under Slightly Acidic Conditions. *Chembiochem : a European journal of chemical biology*, *20*(4), 537–542.

Huang, P. J., Vazin, M., Lin, J. J., Pautler, R., and Liu, J. (2016). Distinction of Individual Lanthanide Ions with a DNAzyme Beacon Array*ACS Sensors,* 1 (6), 732-738
83. Huang, P. J., Lin, J., Cao, J., Vazin, M., & Liu, J. (2014). Ultrasensitive DNAzyme beacon for lanthanides and metal speciation. *Analytical chemistry*, 86(3), 1816–1821.
84. Huang, P. J., Vazin, M., Matuszek, Ż., Liu, J. (2015). A new heavy lanthanide-dependent DNAzyme displaying strong metal cooperativity and unrescuable phosphorothioate effect, *Nucleic Acids Research*, 43(1), 461–469.
85. Huang, P. J., Vazin, M., & Liu, J. (2016). In Vitro Selection of a DNAzyme Cooperatively Binding Two Lanthanide Ions for RNA Cleavage. *Biochemistry*, 55(17), 2518–2525.
86. Zhou, W., Saran, R., Chen, Q., Ding, J., & Liu, J. (2016). A New Na(+)-Dependent RNA-Cleaving DNAzyme with over 1000-fold Rate Acceleration by Ethanol. Chembiochem : *a European journal of chemical biology*, 17(2), 159–163.
87. Huang, P. J., & Liu, J. (2016). An Ultrasensitive Light-up Cu(2+) Biosensor Using a New DNAzyme Cleaving a Phosphorothioate-Modified Substrate. *Analytical chemistry*, 88(6), 3341–3347.
88. Huang, P. J., & Liu, J. (2015). Rational evolution of Cd2+-specific DNAzymes with phosphorothioate modified cleavage junction and Cd2+ sensing. *Nucleic acids research*, 43(12), 6125–6133.
89. Ren, W., Jimmy Huang, P. J., de Rochambeau, D., Moon, W. J., Zhang, J., Lyu, M., Wang, S., Sleiman, H., & Liu, J. (2020). Selection of a metal ligand modified DNAzyme for detecting Ni2. *Biosensors & bioelectronics*, 165, 112285.
90. Gysbers, R., Tram, K., Gu, J., & Li, Y. (2015). Evolution of an Enzyme from a Noncatalytic Nucleic Acid Sequence. *Scientific reports*, 5, 11405.
91. Santoro, S. W., Joyce, G. F., Sakthivel, K., Gramatikova, S., & Barbas CF 3rd (2000). RNA cleavage by a DNA enzyme with extended chemical functionality. *Journal of the American Chemical Society*, 122(11), 2433–2439.
92. Wang, Y., Liu, E., Lam, C. H., & Perrin, D. M. (2018). A densely modified M2+-independent DNAzyme that cleaves RNA efficiently with multiple catalytic turnover. *Chemical science*, 9(7), 1813–1821.
93. Huang, P. J., de Rochambeau, D., Sleiman, H. F., & Liu, J. (2020). Target Self-Enhanced Selectivity in Metal-Specific DNAzymes. *Angewandte Chemie* (International ed. in English), 59(9), 3573–3577.
94. Chiuman, W., & Li, Y. (2007). Simple fluorescent sensors engineered with catalytic DNA 'MgZ' based on a non-classic allosteric design. *PloS one*, 2(11), e1224.
95. Carrigan, M. A., Ricardo, A., Ang, D. N., & Benner, S. A. (2004). Quantitative analysis of a RNA-cleaving DNA catalyst obtained via in vitro selection. *Biochemistry*, 43(36), 11446–11459.
96. Chiuman, W., & Li, Y. (2006). Revitalization of six abandoned catalytic DNA species reveals a common three-way junction framework and diverse catalytic cores. *Journal of molecular biology*, 357(3), 748–754.
97. Chiuman, W., & Li, Y. (2006). Evolution of high-branching deoxyribozymes from a catalytic DNA with a three-way junction. *Chemistry & biology*, 13(10), 1061–1069.
98. Tram, K., Xia, J., Gysbers, R., & Li, Y. (2015). An Efficient Catalytic DNA that Cleaves L-RNA. *PloS one*, 10(5), e0126402.
99. Santoro, S. W., & Joyce, G. F. (1997). A general purpose RNA-cleaving DNA enzyme. *Proceedings of the National Academy of Sciences of the United States of America*, 94(9), 4262–4266.

100. Ordoukhanian, P., & Joyce, G. F. (2002). RNA-cleaving DNA enzymes with altered regio- or enantioselectivity. *Journal of the American Chemical Society*, 124(42), 12499–12506.
101. Schlosser, K., & Li, Y. (2004). Tracing sequence diversity change of RNA-cleaving deoxyribozymes under increasing selection pressure during in vitro selection. *Biochemistry*, 43(30), 9695–9707.
102. Bruesehoff, P. J., Li, J., Augustine, A. J., 3rd, & Lu, Y. (2002). Improving metal ion specificity during in vitro selection of catalytic DNA. *Combinatorial chemistry & high throughput screening*, 5(4), 327–335.
103. Mingqi, W., Huafan, Z., Wei, Z., Yongyun, Z., Afshan, Y., Li, Z., Xiaoqi, Y., Zhuo, T. (2014) In vitro selection of DNA-cleaving deoxyribozyme with site-specific thymidine excision activity, *Nucleic Acids Research*, 42(14), 9262–9269,
104. Schlosser, K., Gu, J., Lam, J. C., & Li, Y. (2008). In vitro selection of small RNA-cleaving deoxyribozymes that cleave pyrimidine-pyrimidine junctions. *Nucleic acids research*, 36(14), 4768–4777.
105. Feldman, A. R., & Sen, D. (2001). A new and efficient DNA enzyme for the sequence-specific cleavage of RNA. *Journal of molecular biology*, 313(2), 283–294.
106. Lam, J. C., Withers, J. B., & Li, Y. (2010). A complex RNA-cleaving DNAzyme that can efficiently cleave a pyrimidine-pyrimidine junction. *Journal of molecular biology*, 400(4), 689–701.
107. Sidorov, A. V., Grasby, J. A., & Williams, D. M. (2004). Sequence-specific cleavage of RNA in the absence of divalent metal ions by a DNAzyme incorporating imidazolyl and amino functionalities. *Nucleic acids research*, 32(4), 1591–1601.
108. Yang, L., Ding, P., Luo, Y., Wang, J., Lv, H., Li, W., Cao, Y., & Pei, R. (2019). Exploration of Catalytic Nucleic Acids on Porphyrin Metalation and Peroxidase Activity by in Vitro Selection of Aptamers for N-Methyl Mesoporphyrin IX. *ACS combinatorial science*, 21(2), 83–89.
109. Perrin, D. M., Garestier, T., & Hélène, C. (2001). Bridging the gap between proteins and nucleic acids: a metal-independent RNAseA mimic with two protein-like functionalities. *Journal of the American Chemical Society*, 123(8), 1556–1563.
110. Saran, R., Chen, Q., & Liu, J. (2015). Searching for a DNAzyme Version of the Leadzyme. *Journal of molecular evolution*, 81(5-6), 235–244.
111. Zhou, W., Ding, J., & Liu, J. (2016). An Efficient Lanthanide-Dependent DNAzyme Cleaving 2'-5'-Linked RNA. *Chembiochem : a European journal of chemical biology*, 17(10), 890–894.
112. Shen, Z., Wu, Z., Chang, D., Zhang, W., Tram, K., Lee, C., Kim, P., Salena, B. J., & Li, Y. (2016). A Catalytic DNA Activated by a Specific Strain of Bacterial Pathogen. *Angewandte Chemie* (International ed. in English), 55(7), 2431–2434.
113. Ali, M. M., Aguirre, S. D., Lazim, H., & Li, Y. (2011). Fluorogenic DNAzyme probes as bacterial indicators. *Angewandte Chemie* (International ed. in English), 50(16), 3751–3754.
114. Hipolito, C. J., Hollenstein, M., Lam, C. H., & Perrin, D. M. (2011). Protein-inspired modified DNAzymes: dramatic effects of shortening side-chain length of 8-imidazolyl modified deoxyadenosines in selecting RNaseA mimicking DNAzymes. *Organic & biomolecular chemistry*, 9(7), 2266–2273.
115. Hollenstein, M., Hipolito, C. J., Lam, C. H., & Perrin, D. M. (2009). A DNAzyme with three protein-like functional groups: enhancing catalytic efficiency of M2+-

independent RNA cleavage. *Chembiochem : a European journal of chemical biology*, 10(12), 1988–1992.

116. Mei, S. H., Liu, Z., Brennan, J. D., & Li, Y. (2003). An efficient RNA-cleaving DNA enzyme that synchronizes catalysis with fluorescence signaling. *Journal of the American Chemical Society*, 125(2), 412–420.

References included in the Help page of DNAmoreDB (Reviews).

1. Breaker R. R. (1997). DNA aptamers and DNA enzymes. *Current opinion in chemical biology*, *1*(1), 26–31.
2. Breaker R. R. (1997). In Vitro Selection of Catalytic Polynucleotides. *Chemical reviews*, *97*(2), 371–390.
3. Breaker R. R. (1997). DNA enzymes. *Nature biotechnology*, *15*(5), 427–431.
4. Li, Y., & Breaker, R. R. (1999). Deoxyribozymes: new players in the ancient game of biocatalysis. *Current opinion in structural biology*, *9*(3), 315–323.
5. Sioud M. (2001). Nucleic acid enzymes as a novel generation of anti-gene agents. *Current molecular medicine*, *1*(5), 575–588.
6. Emilsson, G. M., & Breaker, R. R. (2002). Deoxyribozymes: new activities and new applications. *Cellular and molecular life sciences : CMLS*, *59*(4), 596–607.
7. Silverman S. K. (2005). In vitro selection, characterization, and application of deoxyribozymes that cleave RNA. *Nucleic acids research*, *33*(19), 6151–6163.
8. Silverman S. K. (2008). Catalytic DNA (deoxyribozymes) for synthetic applications-current abilities and future prospects. *Chemical communications (Cambridge, England)*, (30), 3467–3485.
9. Silverman, S. K., & Baum, D. A. (2009). Use of deoxyribozymes in RNA research. *Methods in enzymology*, *469*, 95–117.
10. Schlosser, K., & Li, Y. (2009). Biologically inspired synthetic enzymes made from DNA. *Chemistry & biology*, *16*(3), 311–322.
11. Silverman S. K. (2009). Deoxyribozymes: selection design and serendipity in the development of DNA catalysts. *Accounts of chemical research*, *42*(10), 1521–1531.
12. Silverman S. K. (2010). DNA as a versatile chemical component for catalysis, encoding, and stereocontrol. *Angewandte Chemie (International ed. in English)*, *49*(40), 7180–7201.
13. Hollenstein M. (2011). Expanding the catalytic repertoire of DNAzymes by modified nucleosides. *Chimia*, *65*(10), 770–775.
14. Hollenstein M. (2015). DNA Catalysis: The Chemical Repertoire of DNAzymes. *Molecules (Basel, Switzerland)*, *20*(11), 20777–20804.
15. Chang, D., Zakaria, S., Deng, M., Allen, N., Tram, K., & Li, Y. (2016). Integrating Deoxyribozymes into Colorimetric Sensing Platforms. *Sensors (Basel, Switzerland)*, *16*(12), 2061.
16. Silverman S. K. (2016). Catalytic DNA: Scope, Applications, and Biochemistry of Deoxyribozymes. *Trends in biochemical sciences*, *41*(7), 595–609.
17. Palou-Mir, J., Barceló-Oliver, M., & Sigel, R. (2017). The Role of Lead(II) in Nucleic Acids. *Metal ions in life sciences*, *17*, /books/9783110434330/9783110434330-012/9783110434330-012.xml.
18. Zhou, W., Ding, J., & Liu, J. (2017). Theranostic DNAzymes. *Theranostics*, *7*(4), 1010–1025.

19. Alsaafin, A., & McKeague, M. (2017). Functional nucleic acids as in vivo metabolite and ion biosensors. *Biosensors & bioelectronics*, *94*, 94–106.
20. Zhou, W., Saran, R., & Liu, J. (2017). Metal Sensing by DNA. *Chemical reviews*, *117*(12), 8272–8325.
21. Fokina, A. A., Chelobanov, B. P., Fujii, M., & Stetsenko, D. A. (2017). Delivery of therapeutic RNA-cleaving oligodeoxyribonucleotides (deoxyribozymes): from cell culture studies to clinical trials. *Expert opinion on drug delivery*, *14*(9), 1077–1089.
22. Zhou, W., & Liu, J. (2018). Multi-metal-dependent nucleic acid enzymes. *Metallomics : integrated biometal science*, *10*(1), 30–48.
23. Morrison D., Rothenbroker M. and Li Y. (2018) DNAzymes: Selected for Applications. Small Methods, 2, 1700319.
24. Khachigian L.M. (2019) Deoxyribozymes as Catalytic Nanotherapeutic Agents. Cancer Research, 79(5) 879-888.
25. Kumar, S., Jain, S., Dilbaghi, N., Ahluwalia, A. S., Hassan, A. A., & Kim, K. H. (2019). Advanced Selection Methodologies for DNAzymes in Sensing and Healthcare Applications. *Trends in biochemical sciences*, *44*(3), 190–213.
26. Hwang, K., Mou, Q., Lake, R. J., Xiong, M., Holland, B., & Lu, Y. (2019). Metal-Dependent DNAzymes for the Quantitative Detection of Metal Ions in Living Cells: Recent Progress, Current Challenges, and Latest Results on FRET Ratiometric Sensors. *Inorganic chemistry*, *58*(20), 13696–13708.
27. Hollenstein M. (2019). Nucleic acid enzymes based on functionalized nucleosides. *Current opinion in chemical biology*, *52*, 93–101.
28. Le Vay, K., Salibi, E., Song, E. Y., & Mutschler, H. (2020). Nucleic Acid Catalysis under Potential Prebiotic Conditions. *Chemistry, an Asian journal*, *15*(2), 214–230.
29. Walter, J. G., & Stahl, F. (2020). Aptazymes: Expanding the Specificity of Natural Catalytic Nucleic Acids by Application of In Vitro Selected Oligonucleotides. *Advances in biochemical engineering/biotechnology*, *170*, 107–119.
30. Safdar, S., Lammertyn, J., & Spasic, D. (2020). RNA-Cleaving NAzymes: The Next Big Thing in Biosensing?. *Trends in biotechnology*, S0167-7799(20)30117-7. Advance online publication.
31. Cepeda-Plaza, M., & Peracchi, A. (2020). Insights into DNA catalysis from structural and functional studies of the 8-17 DNAzyme. *Organic & biomolecular chemistry*, *18*(9), 1697–1709.
32. Ma, L., & Liu, J. (2020). Catalytic Nucleic Acids: Biochemistry, Chemical Biology, Biosensors, and Nanotechnology. *iScience*, *23*(1), 100815.
33. Javadi-Zarnaghi, F., & Höbartner, C. (2020). Strategies for Characterization of Enzymatic Nucleic Acids. *Advances in biochemical engineering/biotechnology*, *170*, 37–58.
34. Kosman, J., & Juskowiak, B. (2020). Bioanalytical Application of Peroxidase-Mimicking DNAzymes: Status and Challenges. *Advances in biochemical engineering/biotechnology*, *170*, 59–84.
35. Zimmermann, A. C., White, I. M., & Kahn, J. D. (2020). Nucleic acid-cleaving catalytic DNA for sensing and therapeutics. *Talanta*, *211*, 120709.
36. Balke, D., Hieronymus, R., & Müller, S. (2020). Challenges and Perspectives in Nucleic Acid Enzyme Engineering. *Advances in biochemical engineering/biotechnology*, *170*, 21–35.
37. Xiang, Y., & Lu, Y. (2014). DNA as sensors and imaging agents for metal ions. *Inorganic chemistry*, *53*(4), 1925–1942.

38. Lan, T., & Lu, Y. (2012). Metal ion-dependent DNAzymes and their applications as biosensors. *Metal ions in life sciences*, *10*, 217–248.
39. Rosenbach, H., Victor, J., Etzkorn, M., Steger, G., Riesner, D., & Span, I. (2020). Molecular Features and Metal Ions That Influence 10-23 DNAzyme Activity. Molecules (Basel, Switzerland), 25(13), 3100.
40. Schlosser, K., & Li, Y. (2010). A versatile endoribonuclease mimic made of DNA: characteristics and applications of the 8-17 RNA-cleaving DNAzyme. Chembiochem : *a European journal of chemical biology*, 11(7), 866–879. https://doi.org/10.1002/cbic.200900786

Table S1. JSON/CSV identifiers. Description of the JSON/CSV identifiers used by the API system of DNAmoreDB.

**Identifier**	**Description**
name	Name of the DNAzyme
e	Sequence of the DNAzyme's variable catalytic region
length	Length of the variable catalytic region
metal_ions	List of metal ions/ cofactors required for catalysis
reaction	Reaction catalyzed by the DNAzyme
buffer	Buffer used during the in-vitro selection key selection step
l	Left substrate
r	Right substrate
s	Substrate of the DNAzyme
fgs1	Reacting group/residue 1
fgs2	Reacting group/residue 2
x	Residues other than A, C, G, T, U in the DNAzyme sequence
rp	Reaction product
structures	PDB code(s) of structure(s) related to the DNAzyme entry
main_article_title	Title of the article where the DNAzyme was first reported.
main_article_first_author	Name of the first author listed in the article
main_article_last_author	Name of the last author listed in the article

main_article_mid_authors	Names of other authors
main_article_pub_date	Publication year
reported_in	The DNAzyme can be mentioned in the main text of the publication (“m” indicates main text) or in the supplementary information (“s” indicates the supplement).
kinetics	If there is kinetics data associated with the DNAzyme the value is “y” (yes), otherwise “n”(no).
rate_constant	Reaction rate constant will appear here if available
yield	Product yield will appear here if available
notes	Database curators’ notes on the DNAzyme’s entry
